# Misdiagnosis of carcinoma gall bladder in endemic regions

**DOI:** 10.1186/s12893-022-01793-8

**Published:** 2022-09-18

**Authors:** Kunal Bikram Deo, Mohanasundaram Avudaiappan, Sunil Shenvi, Naveen Kalra, Ritambra Nada, Surinder Singh Rana, Rajesh Gupta

**Affiliations:** 1 Department of Surgical Gastroenterology, Postgraduate Institute of Medical Education and Research (PGIMER), Chandigarh, 160012 India; 2 Present Address: Department of Surgery, B.P. Koirala Institute of Health Sciences (BPKIHS), Dharan, Nepal; 3 Present Address: Department of GI, HPB and Multiorgan Transplant Surgery, Trustwell Hospitals, Bangalore, India; 4 Department of Radiology, Postgraduate Institute of Medical Education and Research (PGIMER), Chandigarh, India; 5 Department of Histopathology, Postgraduate Institute of Medical Education and Research (PGIMER), Chandigarh, India; 6 Department of Gastroenterology, Postgraduate Institute of Medical Education and Research (PGIMER), Chandigarh, India

**Keywords:** Carcinoma gallbladder, Endemic region, Under-diagnosis, Over-diagnosis, Radical cholecystectomy

## Abstract

**Background:**

Incidental carcinoma gall bladder and benign disease in radical cholecystectomy specimen is the cause of concern. We attempted to find out the incidence and reasons thereof in the present study.

**Methods:**

Present study is a retrospective analysis of a prospectively maintained database between July 2002 and July 2019. All patients with a diagnosis of carcinoma gall bladder admitted for surgery were included.

**Results:**

Out of 148 patients, 110 patients had carcinoma gall bladder (CAGB), while 38 patients (25.7%) had incidental carcinoma (under-diagnosis). Radical resection was done in 61/110 (55.4%) patients with clinical CAGB, where 15 (24.6%) patients had benign pathology (“over-diagnosis”). Overdiagnosis was due to xanthogranulomatous cholecystitis (n = 9), chronic cholecystitis (n = 2), tuberculosis (n = 2) and IgG4 related cholecystitis (n = 2). Among 61 patients, a history of weight loss and anorexia were significantly associated with malignancy. Asymmetrical wall thickness was significantly more common in benign mimickers.

Among patients with incidental carcinoma, preoperative ultrasonography reported normal wall thickness of gall bladder in 28 (73.7%), thickened gall bladder wall in 6, and polyp in 3 patients. The resectability rate among incidental carcinoma was 27/38 (71.05%).

**Conclusion:**

Over-diagnosis of the carcinoma gall bladder was present in 24.6%. On the other hand, incidental carcinoma comprised 25.7% of all admissions for carcinoma gall bladder with resectability of 71%.

## Introduction

Carcinoma gallbladder is an aggressive malignancy with a 5-year survival of less than 5% in patients not undergoing curative surgical resection [1]. R0 resection provides the best outcome [2]. Aggressive surgery in achieving R0 resection has been associated with better long-term survival and higher postoperative morbidity and mortality [3, 4].

Even though multiple etiologies have been proposed in the pathogenesis of carcinoma gallbladder, we are still looking for definitive answers [5]. Nonetheless, the most plausible is the dysplasia-metaplasia-carcinoma sequence noted in gallstone carriers [6]. Though earlier studies reported a relationship between the size of gallstone and gallbladder cancer, later studies have failed to prove this correlation [7–9]. Also, the incidence of gallbladder carcinoma is reportedly high in regions endemic to gallstone disease, thus validating the postulation [10].

In the endemic regions of gallstone disease, benign gallbladder pathologies like xanthogranulomatous cholecystitis and thick-walled gall bladder are high [11]. Preoperative characteristics, including biochemical and radiological investigations, might guide in differentiating benign and malignant diseases. However, no modality can accurately distinguish the two entities in the preoperative setting [12]. There remains a high risk of tumor dissemination due to fine-needle aspiration cytology from gall bladder mass, which precludes preoperative tissue diagnosis [13]. The same is the issue with performing simple cholecystectomy for diagnostic purposes, as there is a risk of breach of tumor planes leading to dissemination [14].

Like the high incidence of several benign diseases mimicking malignancy in endemic regions, the frequency of incidental carcinoma gallbladder also parallels the incidence of the carcinoma gall bladder in endemic areas of gall stones disease and carcinoma gall bladder. A true incidental carcinoma gall bladder is detected after histopathological examination of the resected cholecystectomy specimen with no suspicion of malignancy in the preoperative or the intraoperative period. Studies from endemic regions of carcinoma gallbladder in India and Japan show that of all the laparoscopic cholecystectomy specimens, the incidence of the incidental carcinoma gall bladder is 1.9% and 2.3%, respectively [15, 16]. Another study from the United Kingdom reported an incidence of 0.27% of malignancy in the cholecystectomy specimens (n = 1452) [17]. The figures further rise in the presence of other risk factors like a polyp.

Both underdiagnoses, as well as overdiagnosis of carcinoma gall bladder, can adversely affect the outcome and can also invite medico-legal problems. At the same time, it is important to emphasize that simple cholecystectomy for histologically unproven carcinoma gall bladder breaches the tissue planes. If the disease is T1b or more, it can leave metastatic lymph nodes behind. Also, there remains the risk of accidental gall bladder perforation during the procedure, potentially converting a localized disease into a metastatic one. Similarly, it needs to be emphasized during counseling of patients that overtreating benign chronic cholecystitis with radical surgery has the potential of postoperative complications like bile leak and bleeding with a prolonged hospital stay, which may require further intervention.

The present study was conducted to identify the magnitude of under-diagnosis and overdiagnosis of carcinoma gall bladder in regions of high incidence like North India. Possible preoperative measures to identify diagnostic pitfalls and thus avoid radical surgery in benign entities were also studied.

## Materials and methods

We performed a retrospective analysis of a prospectively maintained database of patients admitted at the tertiary care center of North India, a high endemic zone for carcinoma gall bladder and gallstone disease. All patients admitted with the diagnosis of carcinoma gallbladder between January 2002 and July 2019 were included. This study was approved by Institutional Ethics Committee, Postgraduate Institute of Medical Education and Research, Chandigarh (INT/IEC/2019/SPL-1462).

Diagnosis of carcinoma gallbladder was established preoperatively by clinical examination and radiology. Patients who showed no sign of distant metastasis on clinical examination and had no contraindication to major surgery were enrolled for the study. Patients who had undergone cholecystectomy elsewhere and were diagnosed with incidental carcinoma of the gall bladder based upon histopathological evaluation underwent detailed clinical examination to rule out metastatic disease and were also included.

All these patients were subjected to contrast-enhanced computed tomography (CECT) of the abdomen to assess resectability. Patients deemed resectable and operable were admitted. CECT scan was repeated before surgery if the time elapsed from the last CECT to surgery was more than one month. Written informed consent was obtained from all patients. Positron emission tomography- C.T. has been added as a preoperative tool to rule out distant metastasis for the last six years. In patients deemed unresectable or with distant metastasis, tissue diagnosis was obtained, and palliative therapy started.

All patients with clinically resectable disease underwent diagnostic laparoscopy or exploratory laparotomy. Any evidence of distant metastasis or unresectability (involvement of main hepatic artery, main portal vein) was noted. In the presence of these findings, a biopsy was taken, and a palliative procedure was performed if required. All other patients underwent radical cholecystectomy, which included resection of a 2 cm bed of gall bladder or segment IVA and V depending on the depth of hepatic infiltration. Radical surgery was performed to achieve R0 resection. Resection of the common bile duct (CBD) was done if the tumor either directly infiltrated or was close to CBD. In some patients with the bulky hepato-duodenal disease, CBD resection was indicated for complete clearance of the hepato-duodenal ligament. Surrounding organs like the colon, duodenum, or vascular structures like the right hepatic artery were resected when the tumor invaded these structures. If there was back-bleed from the hepatic end of the right hepatic artery, no change in the plan of liver resection was contemplated; otherwise, right hepatectomy along with resection of segment 4b was performed.

All patients had lymph nodal clearance of hepato-duodenal ligament with complete skeletonization of common bile duct, hepatic artery proper, and portal vein, along with the removal of posterosuperior and posteroinferior pancreaticoduodenal lymph nodes, as well as lymph nodes dissection along common hepatic artery and right side of the celiac axis. Aortocaval lymph nodal clearance till anterior spinal ligament was performed in patients with enlarged pancreaticoduodenal lymph nodes. Extended right hepatectomy was performed in tumors infiltrating the porta and deemed resectable. However, all such patients underwent preoperative endoscopic or radiological drainage of bile ducts to reduce serum bilirubin levels below 2 mg% before major liver resection.

Analysis was performed to ascertain any difference between the benign entities and the carcinoma gallbladder. Clinico-demographic features studied were age, gender, history of abdominal pain, duration of jaundice or gastric outlet obstruction, history of weight loss, and presence of comorbidity. Abdominal examination findings were assessed and recorded. Preoperative blood investigations and computed tomography findings were noted in all patients. Among the patients who had undergone surgical exploration, the intraoperative findings (tumor character, adjacent structure involvement, lymph node status, vascular involvement, evidence of distant metastasis) were noted. In addition, histopathology reports were analyzed in patients who had undergone radical surgery.

### Postoperative management

Patients were encouraged for early ambulation, feeding, and aggressive chest physiotherapy. Postoperative morbidity and mortality were recorded. Patients were followed up 2-monthly for 6 months, 3-monthly for 2 years, 4-monthly up to 5 years, and 6- months after that. Node-positive patients received adjuvant chemotherapy and local radiotherapy at the tumor bed. Patients with unresectable disease received palliative therapy after histological confirmation.

### Statistical analysis

The statistical analysis was performed using a statistical package for social sciences 22.0 (SPSS) software. Mean with standard deviation was used for parametric variables, and median with interquartile range (IQR) (25th to 75th percentile) was used for non-parametric variables. The comparison between the two groups was made using Chi-square (X2) test and Fischer Exact test as required for qualitative variables. Independent t-test and Mann–Whitney U were done for quantitative variables to assess the level of significance. A p-value of less than 0.05 was considered significant.

## Results

There were 149 patients admitted with the diagnosis of carcinoma gall bladder during this period. One patient had a postoperative diagnosis of hilar cholangiocarcinoma in the histology report and was excluded. Thus 148 patients were part of the study. 104 (70.3%) females and 44 (29.7%) males were in the study with a mean age of 52.7 ± 10.3 years.

**Fig. 1:**
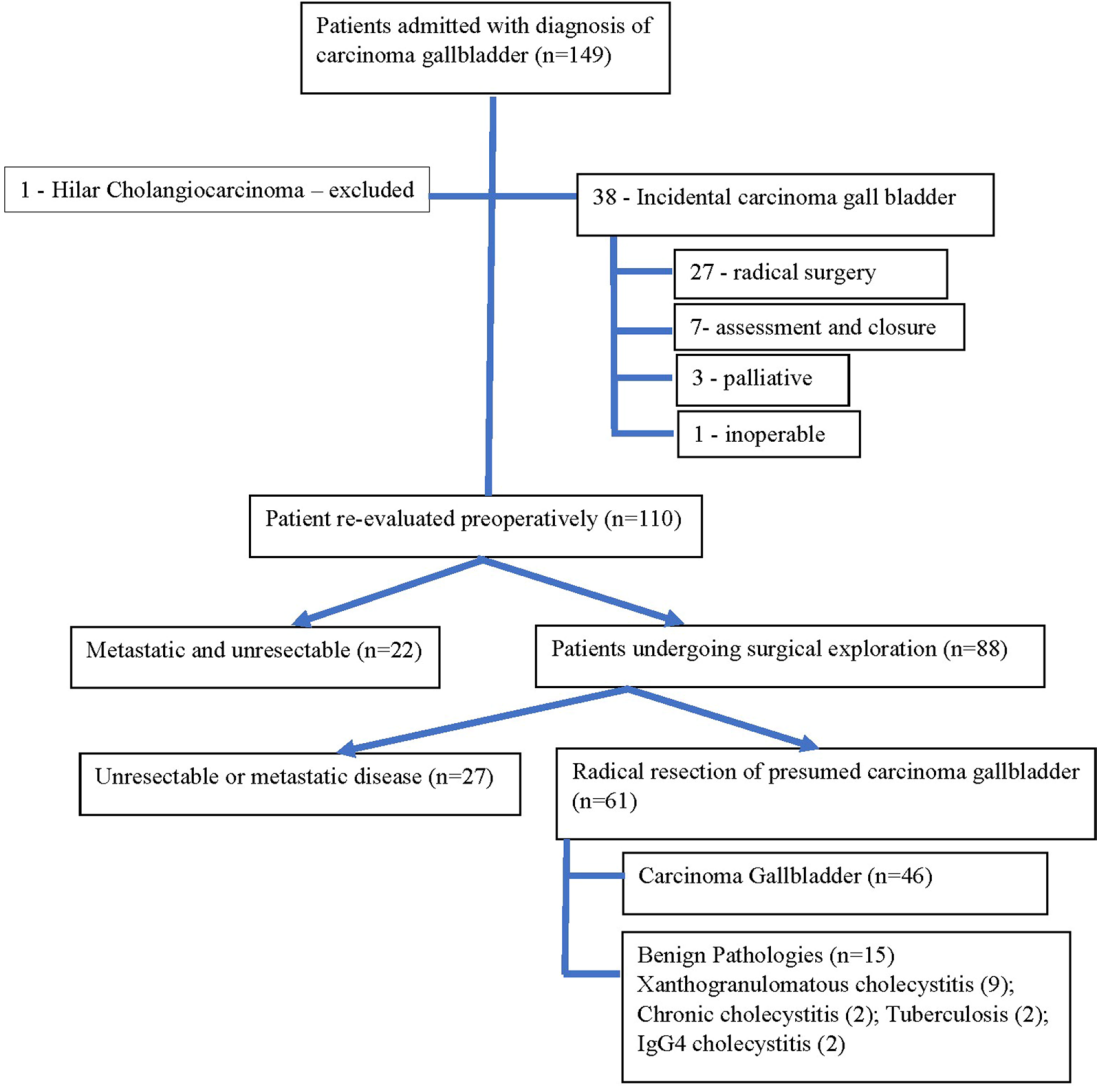
Flow diagram showing the inclusion of the patients and management done

A total of 110 patients were admitted with the clinical diagnosis of resectable carcinoma gall bladder. 22 patients underwent no surgery due to unresectable or metastatic disease detected on repeat preoperative evaluation. The remaining 88 patients underwent surgical exploration with curative intent. Of these, 27 patients did not undergo curative resection based on intraoperative findings, while 61 underwent radical surgery for presumed resectable carcinoma gallbladder. Of 61 patients undergoing radical resection, 15 had benign diseases on the histopathological report (Fig. 1).

When we compared clinical and radiological features between benign and malignant disease, we noted no significant difference in age of presentation or gender distribution. However, the frequency of males was higher in benign pathology compared to malignancy (Table1). We noted a significantly higher incidence of anorexia (p = 0.005) and weight loss (p = 0.04) in patients with malignancy. More smokers were in the benign group than patients with malignancy (Table 1). In preoperative image findings, there was an increased incidence of the mass-forming lesion in the malignant group.

**Table 1:** Comparison of clinical features between the Carcinoma Gallbladder and Benign etiologies

Parameter	Carcinoma Gall bladder (n = 46)	Benign entities (n = 15)	p-value
Mean Age (years)	54.38 ± 9.8	53.46 ± 12.13	0.85
Gender			
Male	12 (26.1%)	7 (46.7%)	0.19
Female	34 (73.9%)	8 (53.3%)	
Abdominal pain	37(80.4%)	14(93.3%)	0.42
Jaundice	12(26.1%)	2(13.3%)	0.48
Weight loss	32 (69.6%)	6 (40.0%)	0.04
Anorexia	28 (60.9%)	2 (13.3%)	0.001
Nausea or vomiting	11 (23.9%)	2 (13.3%)	0.64
Cholangitis	3 (6.5%)	0	0.56
Smoking	3 (6.5%)	4 (26.7%)	0.055
Alcohol	5 (10.9%)	3 (20.0%)	0.39
Comorbidity	18 (39.1%)	2 (13.3%)	0.11
Palpable GB	18 (39.1%)	3 (20.0%)	0.17

In contrast, most benign cases were operated on due to the finding of asymmetrical wall thickness on C.T. images (p = 0.008) (Table 2). Operative findings also revealed mass lesions more frequent in the malignant group and asymmetrically thickened gallbladder walls more frequent in the benign group (p < 0.001) (Table 3). Two patients with asymmetric wall thickening on C.T. Scan had normal wall thickness, and one appeared to have a tumor-like appearance. However, the overlying mucosa was normal on cutting open the operative specimen.

**Table 2:** Comparison of Computed Tomography findings and biochemical parameters between the Carcinoma Gallbladder and Benign etiologies

Parameter	Carcinoma Gall bladder (n = 46)	Benign entities (n = 15)	p-value
Serum albumin > 3.5 gm/dl	29/45	13/15	0.19
Gall stones	14 (30.4%)	9 (60.0%)	0.2
CT tumor morphology			
Thickening	13 (28.3%)	10 (66.7%)	0.008
Mass	33 (71.7%)	5 (33.3%)	
Liver infiltration	26 (56.5%)	9 (60.0%)	0.81
CBD involvement	9 (19.6%)	2 (13.3%)	0.7
Surrounding organ	22 (47.8%)	4 (26.7%)	0.15
Vascular involvement	5 (10.9%)	2 (13.3%)	1.0

**Table 3:** Comparison of intraoperative characters between carcinoma gall bladder and benign pathologies

Parameter	Carcinoma Gall bladder (n = 46)	Benign entities (n = 15)	p-value
Tumor character			
No wall thickening/mass	0	2 (13.3%)*	0.000
Thickening	4 (8.7%)	7 (46.7%)	
Mass	42 (91.3%)	6 (40.0%)	
Liver involvement	18 (39.1%)	8 (53.3%)	0.37
CBD involvement	24 (52.2%)	4 (26.7%)	0.08
Vascular involvement	8 (17.4%)	0	0.17
Organ involved			
Duodenum	7	0	0.49
Colon	8	2	
Duodenum + Colon	7	2	
Overall complications	19 (41.3%)	5 (33.3%)	0.76
Bile leak	8 (17.4%)	5 (33.3%)	0.27
Surgical site infection	10 (21.7%)	4 (26.7%)	0.73
Liver failure	3 (6.5%)	0	0.56
Intra-abdominal sepsis	5 (10.9%)	3 (20.0%)	0.39
Chyle leak	2 (4.3%)	0	1.0
Sepsis with organ failure	3 (6.7%)	0	0.56
Mortality	2 (4.3%)	0	1.0

*Two patients in benign group had neither mass or asymmetrical wall thickening of gall bladder on gross evaluation of resected specimen

Other radiological and operative parameters were comparable between the malignant and benign groups. Resections of adjoining organs like the bile duct, colon, and duodenum were comparable in the two groups. Five patients (33.3%) in benign group had complications like bile leak from liver surface (n = 2), hepaticojejunostomy anastomosis (n = 1), and duodenum (n = 2) leading to intra-abdominal collection (n = 3), surgical site infection (SSI) (n = 4) and lung infection (n = 1). These complications were managed non-surgically. On the other hand, 19 (41.3%) patients in malignant group had complications like bile leak (n = 8), SSI (n = 10), liver failure (n = 3), intra-abdominal sepsis (n = 5), chyle leak (n = 2), sepsis with organ failure (n = 3).

There were mortality in 2/61 (3.3%) patients in the malignant group. One patient had extended right hepatectomy with bile duct resection and main portal vein (MPV) resection with right hemicolectomy and resection of the first part of the duodenum. He had left PV to MPV anastomosis, Roux-en-Y hepatico-jejunostomy, gastrojejunostomy with ileo-transverse anastomosis. The second patient had radical cholecystectomy with CBD resection and had hepatico-jejeunostomy for carcinoma gall bladder with empyema.

**Fig. 2:**
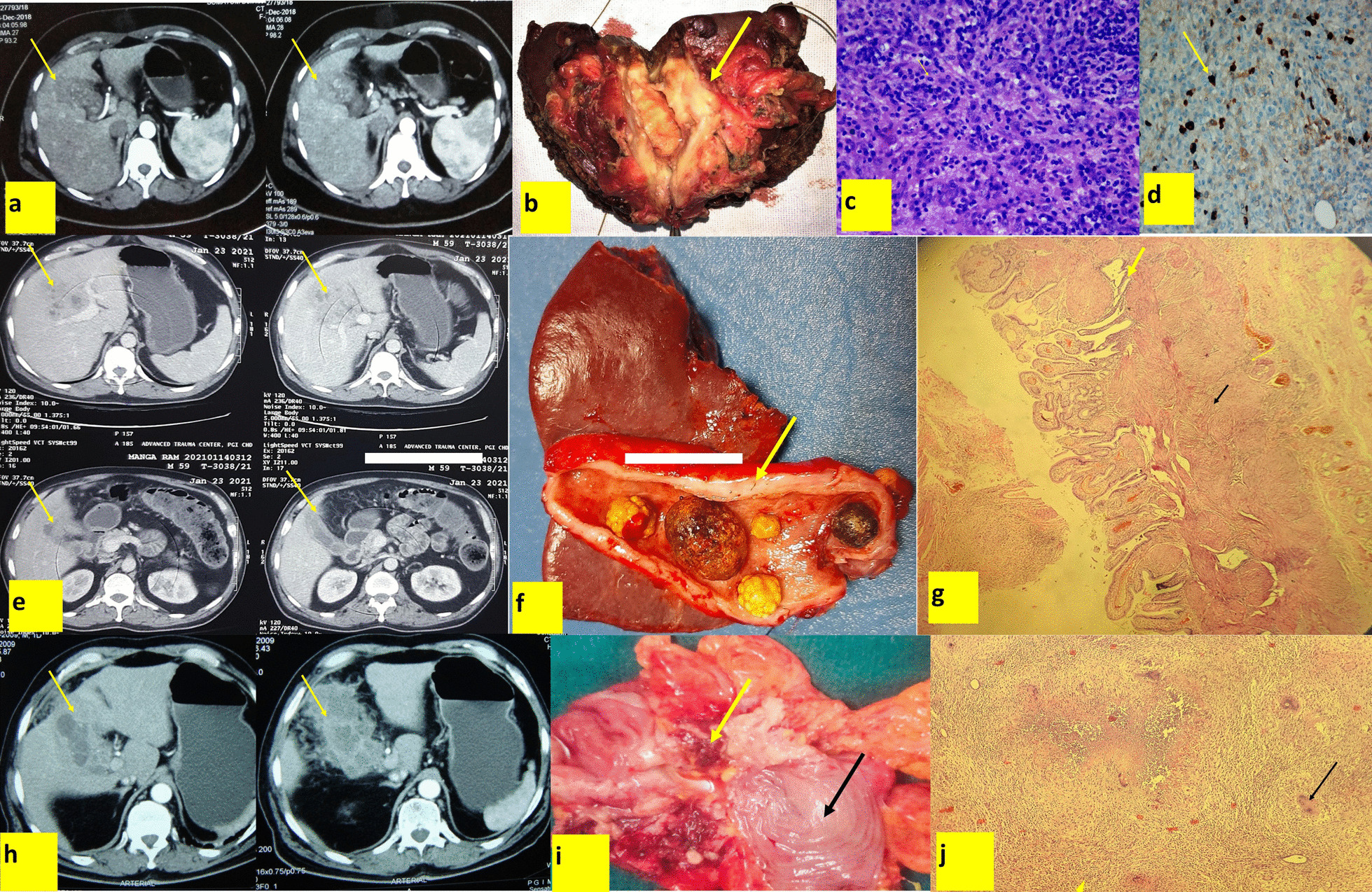
Picture collage showing benign mimickers of the carcinoma gall bladder. **a–d ***IgG4 Cholecystitis*. **a** Contrast-enhanced computed tomogram (CECT) of the abdomen shows irregular heterogeneously enhancing thickened gall bladder wall forming a mass with loss of interface with liver (Yellow arrow). **b** Resected specimen showing irregular thickened gall bladder infiltrating the liver bed (yellow arrow). **c** Photomicrograph (400×) reveals dense infiltration with plasma cells and lymphocytes. These plasma cells have eccentrically placed cartwheel-like nuclei with perinuclear hof. **d** Immunohistochemistry image shows characteristics cell with strong cytoplasmic positivity for IgG4. **e–g ***Xanthogranulomatous Cholecystitis*: **e** CECT of the abdomen shows irregular hypodense thickened gall bladder wall forming a mass with loss of interface with liver (Yellow arrow). **f** Resected specimen showing irregular thickened gall bladder wall (yellow arrow). **g** Photomicrograph (40×) reveals Aschoff-Rokitansky sinus (thick yellow arrow), thickened gallbladder wall with myofibroblastic proliferation (black arrow) with infiltrates of lympho-histiocytes and chronic inflammatory cells (thin yellow arrow). **h–j** *Gall bladder tuberculosis***: h** CECT of the abdomen shows irregular heterogeneously enhancing thickened gall bladder wall with loss of interface with liver (Yellow arrow). **i** Specimen showing multi-visceral resection including gall bladder mass (yellow arrow) with a colon (black arrow) and omentum. **j** Photomicrograph (40×) reveals granulomas with caseous necrosis (yellow arrowhead) and multinucleated giant cells (black arrow)

We also looked at benign mimickers of malignancy in our cohort. We found that xanthogranulomatous cholecystitis (XGC) was the most common benign disease in the resected specimen (n = 9, 69.2%). The other benign conditions were chronic cholecystitis (n = 2), tuberculosis (n = 2), and IgG4-related cholecystitis (n = 2) (Table 4) (Fig. 2). Two patients with benign diseases also presented with jaundice (XGC 1; Tuberculosis 1) (Table 1).

**Table 4:** Demographic, clinical, imaging, and management characteristics of the benign diseases

S no	Age	Gender	Diagnosis	Surgery	Morphology of lesion in CECT Abdomen	Constitutional symptoms Anorexia/ Weight loss	Gallstone	Bile duct resection
1	52	M	Xanthogranulomatous cholecystitis	RC	Mass forming	No	Yes	N
2	49	F	Xanthogranulomatous cholecystitis	RC	Thickening	No	No	Y
3	60	F	Xanthogranulomatous cholecystitis	RC	Mass forming	Yes	Yes	Y
4	48	F	Xanthogranulomatous cholecystitis	RC + segmental colectomy	Thickening	No	No	N
5	59	F	Xanthogranulomatous cholecystitis	RC	Mass forming	Yes	Yes	N
6	70	M	Xanthogranulomatous cholecystitis	RC	Thickening	No	No	N
7	60	M	Xanthogranulomatous cholecystitis	RC + D1 duodendectomy	Thickening	No	Yes	N
8	30	M	Xanthogranulomatous cholecystitis	RC	Thickening	No	No	N
9	60	F	Xanthogranulomatous cholecystitis	RC	Thickening	Yes	Yes	N
10	70	M	Chronic cholecystitis	RC	Thickening	No	Yes	N
11	52	F	Chronic cholecystitis	RC	Thickening	No	Yes	Y
12	34	F	IgG4 related cholecystitis	RC	Thickening	yes	Yes	N
13	48	M	IgG4 related cholecystitis	RC	Mass forming	No	Yes	Y
14	68	M	Tuberculosis	RC + D1 duodenectomy + segmemtal colectomy	Mass forming	Yes	Yes	N
15	42	F	Tuberculosis	RC	Thickening	Yes	Yes	Y

*RC* Radical cholecystectomy

On the other hand, there were 38 (25.7%) patients with incidental carcinoma. Of these 38 patients, 24 (63.2%) had undergone open cholecystectomy, while the rest had a laparoscopic cholecystectomy. Preoperative ultrasound reports in 28/38 (73.7%) patients had revealed only gall stones. In contrast, gall bladder wall thickening and polyp were reported in 6 and 3 patients, and mass in the gall bladder in one patient. Other abnormal findings reported on preoperative ultrasound included CBD dilatation in one and mucocele in two patients. Complete histopathological report of the cholecystectomy specimen was available in 30/38 patients, with 22/30 (73.3%) having ≥ T2 disease (Table 5). Among these 38 patients, 27 (71.1%) underwent curative surgical resection in our center, while 3 underwent the palliative procedure. The rest of 8 patients (21%) had metastatic disease.

**Table 5:** Pathological staging of the cholecystectomy specimen in the incidental carcinoma gall bladder group

Pathological staging	Lap cholecystectomy	Open cholecystectomy
Normal	0	1
pT1	3(2)	4 (2)
pT2	4(3)	11 (8)
pT3	2 (1)	5 (4)

Numbers in parenthesis indicate the number of patients of that particular stage who had normal ultrasound report

## Discussion

Gallbladder cancer is the most common biliary tract cancer [18]. It has variable incidence across the globe, with high incidence in Asian countries. In India, the incidence of carcinoma gall bladder is estimated to be 22 per 100,000 population [19]. As opposed to this, the incidence is less than 3 per 100,000 in most North European and North American countries [19]. Though not proven comprehensively, this large difference may be secondary to endemic regions of gallstone diseases, genetic predisposition, or chronic infection of Salmonella typhi or Helicobacter pylori [20]. Our center is a tertiary care facility located in the endemic belt of the carcinoma gall bladder.

Clinically it can become difficult to differentiate between benign and malignant pathology, as noted in our study; however, the presence of anorexia and weight loss were significantly more likely in malignant than the benign disease. The presence of such a history is valuable in the clinical diagnosis of malignancy since there are no reliable tumor markers for carcinoma gall bladder. Computed tomography is an important modality in differentiating between benign and malignant etiology. In the present series, we noted mass formation in a significantly greater number of patients with malignancy compared to benign disease. Our study also revealed more chances of benign disease in patients with asymmetric thickening of the gall bladder. In our previous report on resected cases of the carcinoma gall bladder, we had reported that mass in the gall bladder was the commonest finding on C.T. scan in the resectable carcinoma gall bladder (55%) followed closely by diffuse or focal thickening in 45% [21]. Our results reveal that gall bladder wall thickening on C.T. scan needs further correlation with the clinical presentation like loss of weight and appetite. This is important since these patients are unlikely to undergo FNAC.

In the present series, xanthogranulomatous cholecystitis (XGC) was the commonest mimicker of malignancy. Its incidence parallels the burden of gallstone disease [22]. The incidence of XGC was 8.9% in India [23]. Adjacent organ involvement is also commonly seen in this entity due to the spread of inflammation to surrounding organs [11, 12]. In addition, wide variation in the morphological features also adds to the difficulty in its differentiation from malignancy [11]. One of the patients with XGC in the present series presented with jaundice, and three patients with weight loss. Radiology revealed asymmetric thickening in 6 (66.6%) and mass in 3 patients (33.3%). The adjacent structures were also resected for three patients based on preoperative imaging.

Chronic cholecystitis is another common benign condition that can mimic carcinoma gall bladder. Chronic cholecystitis mostly presents diffuse gallbladder wall thickening due to repeated inflammation and localized thickness in some patients, posing a diagnostic dilemma [24]. In an earlier report describing computed tomography findings in patients with cholecystitis, the authors reported a wide variety of findings about the contour of thickening, the extent of thickening, and the pattern of enhancement [25]. Carcinoma gallbladder can present with as many as eight enhancement patterns. The commonest enhancement pattern is hyperdense in the arterial phase and isodense in the venous phase, which is seen in 45.7% of patients. On the other hand, classical enhancement patterns for chronic cholecystitis include iso-attenuation of the inner layer during both phases of computed tomography seen in 89.4%. The same enhancement pattern is also observed in 14.2% of patients with carcinoma gallbladder leading to overlapping [24].

The present series of benign mimickers also include two cases of IgG4-related cholecystitis. One presented with asymmetrical wall thickening and the other with gall bladder mass. IgG4 has been reported earlier also as a mimicker of carcinoma gall bladder [26–28]. Two of the benign cases were tuberculosis in our series. One of the two cases of Gallbladder tuberculosis in the present series presented with jaundice due to bile duct involvement. Both these patients had a history of weight loss. The preoperative C.T. indicated a mass forming lesion in one and asymmetrical thickening in another patient with loss of interface with the liver. Importantly, none of these patients had other focus or history of tuberculosis. All these features make differentiating gall bladder tuberculosis from carcinoma gall bladder extremely difficult. One of these patients with jaundice underwent bile duct resection also. In a case series of 7 patients with gallbladder tuberculosis [29], C.T. morphology of thick wall was most common while micronodular type (n = 2) and mass forming type (n = 1) were also noted. Positron emission tomographic (PET) scans show increased metabolic activity, further complicating preoperative diagnosis [30, 31]. The presence of other foci of abdominal tuberculosis in these patients can be helpful.

The resection of adjacent organs, including the bile duct in benign disease, resulted in morbidity in almost one-third of all resected benign cases in the present series. The extent of resection was dictated by the C.T. scan and intraoperative findings for these mimickers of the carcinoma gall bladder. One has to make it clear to the patient at the beginning of treatment that performing simple cholecystectomy can violate tissue planes in the likelihood of the patient having carcinoma gall bladder and was not the right choice [32]. Therefore, the surgeon must inform the patient of the possibility of the lesion turning out to be a benign disease, especially if gall stones are not present in the gall bladder.

One-third of patients in the present series were referred to our facility as incidental carcinoma. We have noted two dominant referral patterns for incidental carcinoma gall bladder. One subset of patients is referred following a surprise pathology report of malignancy in a cholecystectomy specimen. Another subset of patients had undergone cholecystectomy but was not properly followed with the histological report and later presented with features of advanced or metastatic disease.

Laparoscopic cholecystectomy is performed in most centers based on ultrasound reports of gall stones and liver function tests. When we reviewed the preoperative ultrasound reports, it was apparent that the ultrasound report was suspicious in 10 patients and was likely overlooked (6 patients were reported with gall bladder wall thickening, 3 patients with a polyp, and one with mass). While in the remaining 28 patients, only gall stones were reported. This is likely in infiltrating type of carcinoma gall bladder where the sensitivity of ultrasound can be as low as 12.5%, as noted earlier. In addition, the presence of T1 disease (seen in 7/30 histopathology reports) could also contribute to difficulty in diagnosis unless special efforts are put to pick up subtle abnormalities like focal wall thickening and changes in echo pattern of layers of gall bladder wall [33]. Therefore, the authors recommend that every effort must be made by sonologist to carefully look for concomitant features of underlying malignancy in gall stone disease, and any suspicious feature must be highlighted and surgeons alerted to the possibility of underlying malignancy. In addition, we strongly recommend that the operating surgeon examine the histopathology report of all patients undergoing cholecystectomy for gall stones.

Twenty-seven of the 38 patients (71.1%) with incidental carcinoma gall bladder underwent successful re-resection. Seven patients had a peritoneal disease, two had para-aortic lymph node involvement, and two cases had locally advanced disease with vascular involvement. Shukla et al. [34] also reported a similar rate (71%) of curative surgery in the incidental carcinoma gall bladder. The occurrence of metastatic disease following cholecystectomy for missed carcinoma gall bladder is unfortunate but a grim reality. One reason for intra-abdominal metastasis in these patients can be inadvertent gall bladder perforation and spillage of bile in the peritoneal cavity. Therefore, surgeons need to make every effort to limit the spillage in the peritoneal cavity. As a rule, the operating surgeon should carefully examine all such operative specimens with intraoperative perforation and bile spillage for any suspicious lesion. In case of a suspicious area, the specimen should be sent for a frozen section and copious peritoneal lavage instituted. Depending on the extent of spillage in the peritoneal cavity, even midline laparotomy may be required to deal with spillage effectively, considering that this is the only real chance for the surgeon to salvage the tumor spread in the peritoneal cavity.

In conclusion, the high prevalence of gallstone disease in the endemic region of carcinoma gall bladder results in a significant proportion of benign conditions undergoing major resection (24.6%). Also, an alarming percentage of patients presented with missed carcinoma gall bladder (25.7%). Considering the high rate of metastatic disease (21%) in incidental carcinoma, all attempts should be made to prevent gall bladder perforation and minimize bile spillage during all laparoscopic cholecystectomies. In addition, we recommend that all gallbladder specimens with perforation should be carefully examined on the table by the surgeon to rule out any suspicious area for malignancy and if the required frozen section should be performed. The high incidence of overdiagnosis in our series was due to the considerable overlap of clinical and radiological features between benign conditions and carcinoma gall bladder. In such doubtful situations, authors recommend erring on the side of radical surgery and counseling patients regarding the possibility of benign histology on histopathology reports. An aggressive search for accurate tumor markers, improvement in preoperative imaging, and good history can help reduce diagnostic pitfalls.

## Data Availability

The datasets used and/or analyzed during the current study are available from the corresponding author on reasonable request.
